# Low redundancy in seed dispersal within an island frugivore community

**DOI:** 10.1093/aobpla/plv088

**Published:** 2015-07-18

**Authors:** Kim R. McConkey, Donald R. Drake

**Affiliations:** 1 School of Biological Sciences, Victoria University of Wellington, PO Box 600, Wellington, New Zealand; 2 Department of Botany, University of Hawai‘i at Manoa, 3190 Maile Way, Honolulu, HI 96822, USA; 3 Present address: School of Natural Sciences and Engineering, National Institute of Advanced Studies, Indian Institute of Science Campus, Bangalore, India

**Keywords:** Ecological redundancy, flying foxes, frugivore, fruit bats, functional extinction, Pacific islands, *Pteropus*, seed dispersal

## Abstract

The low species diversity that often characterizes island ecosystems could result in low functional redundancy within communities. Flying foxes (large fruit bats) are important seed dispersers of large-seeded species, but their redundancy within island communities has never been explicitly tested. In a Pacific archipelago, we found that flying foxes were the sole effective disperser of 57 % of the plant species whose fruits they consume. They were essential for the dispersal of these species either because they handled >90 % of consumed fruit, or were the only animal depositing seeds away from the parent canopy, or both. Flying foxes were especially important for larger-seeded fruit (>13 mm wide), with 76 % of consumed species dependent on them for dispersal, compared with 31 % of small-seeded species. As flying foxes decrease in abundance, they cease to function as dispersers long before they become rare. We compared the seed dispersal effectiveness (measured as the proportion of diaspores dispersed beyond parent crowns) of all frugivores for four plant species in sites where flying foxes were, and were not, functionally extinct. At both low and high abundance, flying foxes consumed most available fruit of these species, but the proportion of handled diaspores dispersed away from parent crowns (quality) was significantly reduced at low abundance. Since alternative consumers (birds, rodents and land crabs) were unable to compensate as dispersers when flying foxes were functionally extinct, we conclude that there is almost no redundancy in the seed dispersal function of flying foxes in this island system, and potentially on other islands where they occur. Given that oceanic island communities are often simpler than continental communities, evaluating the extent of redundancy across different ecological functions on islands is extremely important.

## Abstract (precis)

Flying foxes (large fruit bats) play a vital function in dispersing seeds within a Pacific archipelago. More than 75% of plant species eaten by flying foxes, and that had large fruits, were not dispersed effectively by any other animal. Even when plant species had alternative dispersers, these frugivores were often unable to compensate for flying foxes when their role was limited by low numbers. The low functional redundancy within this island system may be characteristic of other island communities which typically have very low species diversity.

## Introduction

Resilience to disturbance is greatest in ecosystems that have high species diversity because of the buffering effect diversity can have on function (Mayfield *et al*. 2010; Dalerum *et al*. 2012; Reich *et al*. 2012). When multiple species perform a given ecosystem function, there is redundancy within the system, and the function may be fully or partially maintained following perturbations in species populations (Dalerum *et al*. 2012). As ecosystems lose species, however, associated declines in functional redundancy increase the vulnerability of these ecosystems to further change (Reich *et al*. 2012). Islands are characterized by inherently low species diversity compared with continents (MacArthur 1965; Whittaker and Fernández-Palacios 2007), and they have been disproportionately further depleted by human-mediated extinctions (e.g. Olson and James 1982; Steadman *et al*. 1991; Steadman 2006). Hence, current island ecosystems might exhibit especially low functional redundancy, which makes the ongoing human-mediated disturbances to them (Brooks *et al*. 2002; Whittaker and Fernández-Palacios 2007) a serious threat to their stability (Cox *et al*. 1991; Traveset *et al*. 2012). An alternative view is that island systems may be somewhat buffered against low functional redundancy because island species are often generalists, or even super-generalists, in their diet and habitat use (Banack 1998; Olesen *et al*. 2002). Hence, understanding the vulnerability of island species to a lack of functional redundancy is complicated, but important, to ensure that functional ecosystems are maintained.

Fruit bats in the family Pteropodidae are effective seed dispersers throughout the Old World tropics (Richards 1990; Rainey *et al*. 1995; Banack 1998; Hodgkison *et al*. 2003; Bollen *et al*. 2004; Nyhagen *et al*. 2005). Flying foxes (*Pteropus* spp.) are predominantly found on islands, with a distribution stretching from the coast of East Africa, through tropical Asia, to Polynesia. Simplified frugivore communities exist on many of these islands, with especially low diversity in the tropical Pacific (Steadman 2006). Here, flying foxes, many species of which declined following human discovery of the islands (Steadman 2006), have generalist diets (Banack 1998) and are often regarded as ‘keystone’ seed dispersers, particularly for large-seeded plants, because of a relative lack of other large frugivores (Cox *et al*. 1991; Rainey *et al*. 1995; McConkey and Drake 2002). In many places, the only extant alternative dispersers of large-seeded species are pigeons, whose role is limited by their gape size (Meehan *et al*. 2002; McConkey *et al*. 2004*a*), and non-volant animals (e.g. rats and crabs) that may sometimes disperse seeds (Lee 1985, 1988; O'Dowd and Lake 1991; McConkey *et al*. 2003; Pérez *et al*. 2008; Shiels and Drake 2011) but are unlikely to be functionally similar to flying foxes. Hence, this community might be expected to show very low redundancy in seed dispersal, especially for large-seeded fruit (Meehan *et al*. 2002). With ∼80 % of plant species displaying zoochorous dispersal mechanisms on some islands (Fall *et al*. 2007), reductions to flying fox populations could have widespread effects on the ecosystem.

The abundance of flying foxes relative to the number of food-bearing trees has a direct, non-linear relationship with their function as seed dispersers (McConkey and Drake 2006). When flying fox abundance is low, the animals remain within the fruiting plant to feed, dropping all unswallowed seeds (flying foxes cannot swallow seeds >4 mm) directly underneath. As fruiting plants fill with animals, feeding territories become fully occupied, forcing any additional flying foxes to ‘raid’ the occupied trees for fruit, which they take to another tree to consume. Only ‘raiders’ are likely to disperse large seeds beyond parent crowns (Richards 1990); consequently, flying foxes become functionally extinct as seed dispersers once their abundance drops below a habitat-specific threshold at which ‘raiding’ begins (Richards 1990; McConkey and Drake 2006). If the frugivore community has low functional redundancy, then declines in flying fox populations resulting from habitat loss, introduced predators, hunting (Wiles *et al*. 1997; Brooke 2001; Jenkins *et al*. 2007; Palmeirin *et al*. 2007) and cyclones (Pierson *et al*. 1996; McConkey *et al*. 2004*b*) will have large consequences for the island ecosystems in which they occur. Given that >70 % of island flying fox species are threatened, near threatened or lacking sufficient information for assessment (IUCN 2014), testing their functional redundancy as dispersers on islands has become urgent.

Our aim was to assess the functional redundancy of an island population of flying foxes in the seed dispersal of their main food plants. We addressed the following hypotheses: (i) relative to other dispersers, flying foxes disperse a disproportionately high proportion of seeds of large-seeded species (defined here as dispersal quantity); (ii) flying foxes disperse a higher proportion of handled seeds away from parent crowns than other dispersers, and mean dispersal distances are greater (defined here as dispersal quality); (iii) flying foxes have greater ‘seed dispersal effectiveness’ (SDE = quantity × quality, Schupp *et al*. 2010) for large diaspores than other dispersers and (iv) total SDE of plants is reduced at sites where flying foxes are functionally extinct.

## Methods

Research was conducted in the Vava‘u archipelago of Tonga in Western Polynesia between June 1999 and June 2001. The archipelago includes 64 islands (total land area 143.3 km^2^; range: <1–96 km^2^) spread over ∼750 km^2^ of ocean. The vegetation was mainly mature rain forest 20–25 m tall (described in Franklin *et al*. 1999). The frugivore community consisted of the insular flying fox *Pteropus tonganus* (body mass averages 428 g; this and following measurements are taken from Gibbons and Watkins 1982; Meehan *et al*. 2002; del Hoyo *et al*. 2013), Pacific pigeon *Ducula pacifica* (395 g), three small dove species (*Ptilinopus perousii* (90 g), *P. porphyraceus* (110 g) and *Alopecoenas stairi* (syn. *Gallicolumba stairi* (Gray, 1856), 171 g)), four even smaller passerines that are at least partially frugivorous (*Pycnonotus cafer* (34 g), *Aplonis tabuensis* (60 g), *Foulehaio carunculatus* (31 g) and *Lalage maculosa* (30 g)), three rat species (*Rattus norvegicus* (215 g), *R. rattus* (140 g) and *R. exulans* (92 g)), crabs (*Coenobita* spp.; measurements not available) and a rarely observed iguana (*Brachylophus fasciatus*; 160 g).

### Identifying alternative dispersers for flying fox-dispersed seeds

**Figure 1.**
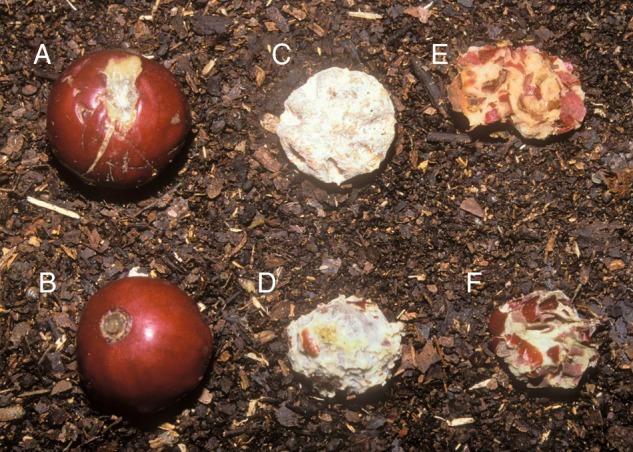
Fruit and diaspores of *Pleiogynium timoriense* showing signs of flying fox feeding. (A) Fruit handled by flying fox. Triangular tooth mark is visible; (B) entire unhandled fruit; (C) day-old and (D) fresh endocarp with most flesh chewed off; (E) day-old and (F) fresh spat out wads of pulp.

Over a 2-year sampling period, we identified consumers of different plant species through direct observations and feeding signs left on fruit and seeds. We conducted systematic and opportunistic searches for handled fruit and dispersed seeds within the forest and under daytime bat roosts, as part of seed dispersal studies on flying foxes (McConkey and Drake 2006) and Pacific pigeons (McConkey *et al*. 2004*a*; Meehan *et al*. 2005). Flying foxes can disperse seeds by swallowing and defecating (seeds <4 mm in diameter) or by carrying the fruit away from the parent plant to eat elsewhere and subsequently dropping the unconsumed seeds (Richards 1990). Diaspores (single- or multi-seeded dispersal units) handled by flying foxes were identified by distinctive impressions left in the pulp (triangular teeth marks in the pulp adhering to the seed), by corresponding wads of spat out pulp or by recovery from bat faeces (Fig. 1). Since the searches were conducted under the mature rain forest canopy, there might be a bias to finding species with larger diaspores. However, smaller-seeded species were deposited in faeces under roosts or fruiting trees, and the easily recognizable wads of fruit pulp also enabled their identification. Hence, we believe the bias to be small.

We classified species with fruits eaten by flying foxes as: (i) ‘commonly-eaten’ species, having multiple records of flying fox foraging and dispersal; (ii) ‘rarely-eaten’ species, which were plants that were found often but had only one or two records of feeding by flying foxes; these bore bat-teeth marks but most of the pulp remained and (iii) ‘damaged’ species, having fruits whose seeds were eaten by flying foxes. These might still be dispersed by flying foxes if carried away from the parent plant for consumption but dropped before the seed was destroyed; this was observed during the study.

Other frugivores also leave unique impressions on diaspores: pigeon-dispersed diaspores are defecated in identifiable scats or regurgitated (single clean diaspores), crabs leave linear claw marks in the pulp and rodents leave incisor marks in the pulp or diaspores (we were unable to distinguish rat species and discuss them collectively). During the study period, we recorded all diaspores regurgitated (*n* = 10 samples) by pigeons or found in their scats (*n* = 67; McConkey *et al*. 2004*a*), in rat husking stations (*n* = 13 720 diaspores; McConkey *et al*. 2003) or handled by crabs (*n* = 140 diaspores).

For small seeds (defined below), the feeding sign of doves and passerines (collectively referred to as ‘small birds’ hereafter) is probably not always distinguishable from pigeons. Our list of dispersed species for these birds was generated from both direct observations and pigeon-dispersed species that had diaspores small enough (<14 mm wide) to be swallowed by doves, which were the largest birds within this category (Meehan *et al*. 2002).

To investigate the different contributions of frugivores according to diaspore size, we classified diaspores into four size categories based on the ability of birds and flying foxes to swallow them (cf. Meehan *et al*. 2002): *small* (S <4 mm), which can be swallowed by all dispersers, except crabs; *medium* (M = 4–13 mm), which can be swallowed by small birds and pigeons; *large* (L = 14–27 mm), which can be swallowed only by pigeons and *extra large* (XL >27 mm), which cannot be swallowed by any dispersers.

### Measuring quantity, quality and SDE

We conducted intensive seed dispersal studies on a selection of the most common species in the diet of flying foxes (*n* = 83 plants of 14 species; McConkey and Drake 2006). All had ripe fruit containing diaspores too large for flying foxes to swallow and were easily detected on the forest floor. Two species had diaspores that were small enough to be swallowed by some small birds (M), seven could be swallowed only by pigeons (L) and five exceeded the gape of any frugivore (XL) (Table 1).

**Table 1.** Plant species consumed by flying foxes in the Vava’u Islands of Tonga during the study period. Species are divided into categories of increasing seed width: small (S < 4 mm), which can be swallowed by flying foxes and all birds; medium (M = 4–13 mm), which can be swallowed by small birds and pigeons; large (L = 14–27 mm), which can be swallowed only by pigeons;  and extra large (XL > 27 mm), which cannot be swallowed by anything (cf. Meehan *et al*. 2002). Alternative dispersers are taken from our observations as well as previously published records and seed size (Meehan *et al*. 2002): P, pigeon; S, small birds; R, rats; C, crabs; when an animal acts as a predator, it is in small letters, so Rr means rats act as both dispersers and predators. A question mark means possible disperser. Abbreviations after the species name indicate species used for more intensive studies. ^1^Species is not commonly consumed by flying foxes; ^2^syn. *Planchonella grayana*; ^3^species not in italics have seeds that are eaten by flying foxes. In the case of *Maniltoa grandiflora*, we recorded a single seed that had minimal damage and had been dispersed away from the canopy. Hence, these species might still be effectively dispersed, but both were uncommon in the diet and could not be fully assessed.

Seed width (mm)	Have other dispersers		No other dispersers	
	Species	Eaten by	Species	Eaten by
Small (<4)	*Ficus* 4 spp.,	P S R?	*Melodinus vitiensis*	
			*Passiflora aurantia*^1^	R?
Medium (4–13)	*Micromelum minutum*	P S r	*Pouteria grayana*^2^ PG	r c
	*Morinda citrifolia*	P		
	*Diospyros elliptica* DE	P S r c		
	Podocarpus pallidus^1,3^	P		
	*Vavaea amicorum*^1^	P S		
	*Jasminum didyum*^1^	P S		
Large (14–27)	*Syzygium clusiifolium* SC	P	Maniltoa grandiflora^1,3^	r
	*Termialia litoralis* TL	P		
	*Chionanthus vitiensis* CV	P Rr		
	*Pleiogynium timoriense* PTi	P Rr C		
	*Syzygium dealatum* SD	P		
	*Faradaya amicorum* FA	P		
	*Guettarda speciosa* GS	P r		
	*Ochrosia vitiensis*^1^	P		
	*Canarium harveyi*^1^	P r		
	*Hernandia nymphaeifolia*^1^	P		
Extra large (>27)	*Mangifera indica* MI	r C	*Burckella richii* BR	r
	*Pandanus tectorius* PTe	Rr C	*Terminalia catappa* TC	r
	*Neisosperma oppositifolium*	Rr C		
	*Inocarpus fagifer* IF	r Cc		

Studies were conducted on eight islands over 15 independent visits (5–9 trees per visit). From each plant's trunk to 45 m beyond the crown edge, four 2-m-wide transects were checked for freshly dispersed (handled) seeds and fallen fruits every morning for 3–5 consecutive days. If a fruiting conspecific plant was found along this transect or near to the edge, the transect extended halfway between the trees. We ensured that each tree had at least two transects extending the full 45 m, and the majority had three or four. We identified the animal responsible for the handled seeds by their feeding sign. Seed densities (seeds m^−2^ day^−1^) were calculated for seeds dispersed under the plant crown and at 1-m increments from the crown edge. We used the seed density to calculate the total number of diaspores dispersed by each frugivore at each 5-m distance increment; total seed fall was the product of the recorded seed density at that distance category and the total area around the tree at that distance (calculated using the canopy radii and the formula of an ellipse).

Seed dispersal effectiveness (Schupp *et al*. 2010) integrates the quantity and quality components of seed dispersal. We defined ‘quantity’ as the proportion of all seeds that had been handled and dispersed by a particular disperser species and ‘quality’ as the proportion of these seeds that were dispersed at least 5 m from the parent canopy by the same species. The 5-m cut-off was chosen because whole, unhandled fruits frequently bounced or rolled ≤5 m from the edge of the parent crown, suggesting that handled fruits found in the same range could have been dropped from within the fruiting crown. Our measure of dispersal quality (and therefore SDE) is incomplete since we do not know the recruitment potential of the seeds dispersed at different distances. Here, we base quality on the minimum assumption that seeds dispersed away from parent crowns are more likely to establish than seeds dispersed under the crown, and this Janzen–Connell effect has shown to be a common scenario for most tropical trees (Swamy and Terborgh 2010). We calculated SDE for plant species for which we had data for three or more individual plants.

### Functional extinction of flying foxes and SDE

The effectiveness of flying foxes as seed dispersers is non-linearly related to their abundance since they become functionally extinct once their abundance drops below a habitat-specific threshold (McConkey and Drake 2006). Flying foxes were abundant in the island group during our study, but local abundance at any site varies temporally and spatially as individuals track fruit supplies around the islands (McConkey and Drake 2007). We used this natural variability to compare SDE values for flying foxes in sites where they were at low abundance relative to food availability (i.e. functionally extinct) with sites where their relative abundance was high. We also used the SDE landscape (Schupp *et al*. 2010) to investigate the relative contributions of other frugivores at these sites and determine whether they compensated for flying fox loss. Site categories used followed McConkey and Drake (2006).

### Statistical analysis

To evaluate whether the SDE of flying foxes was significantly altered by their abundance, we compared SDE values of all species from low- and high-abundance sites using a *t*-test. *t*-Tests were also used to compare the quantity and quality values in low- and high-abundance sites for one species, *Pleiogynium timoriense*, for which we had >10 studied trees in each category. We used the conservative approach of identifying non-overlapping confidence intervals to evaluate differences in mean dispersal distances among species. *Z*-tests were used to compare the proportion of diaspores dispersed under conspecific crowns by flying foxes, with other frugivores. We only used trees studied at sites where flying fox abundance was high (i.e. where they were not functionally extinct) to determine the proportions dispersed by flying foxes. Statistical analyses were done using Sigmastat 3.5.

## Results

### Alternative dispersers for flying fox-dispersed seeds

**Figure 2.**
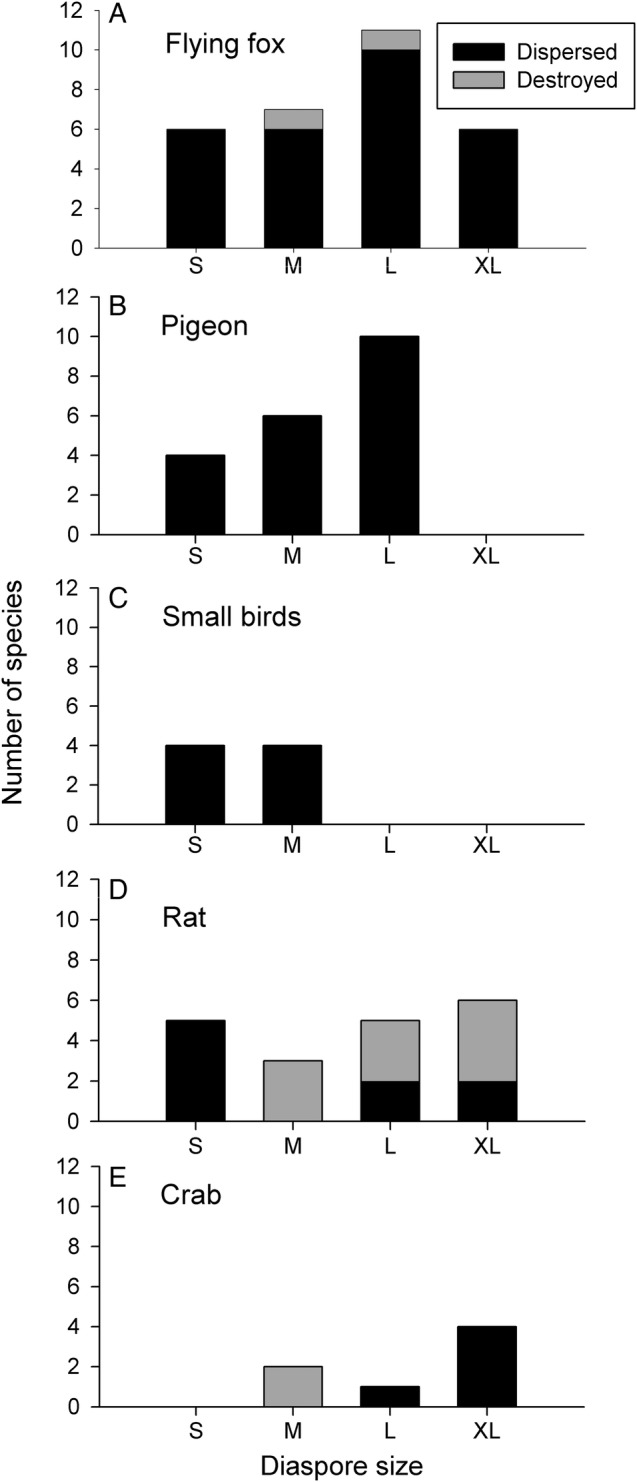
Overlap in seed dispersal services provided by flying foxes and other fruit-eating animals in Tonga. The figure shows the number of plant species that are dispersed (black) or destroyed (grey) by (A) flying foxes and how many of these species are also consumed by other animals (B–E). Dispersed species are arranged into four categories of diaspore size: small (S < 4 mm), which is the gape limit of flying foxes; medium (M = 4–13 mm), which is the gape limit of small birds; large (L = 14–27 mm), which is the gape limit of pigeons and extra large (XL > 27 mm) cannot be swallowed by anything (cf. Meehan *et al*. 2002).

Fruits of 30 plant species being handled by flying foxes at the study site were recorded (22 commonly-eaten species, 8 rarely-eaten species, of which 2 had the seeds partially eaten) and 6 of these had no alternative dispersers. Six of the consumed plant species had small diaspores and four of these had several alternative dispersers (Table 1, Fig. 2). One of the seven species with medium diaspores (*Planchonella grayana* H.St.John) was dispersed only by flying foxes; its seeds were encased in large, hard fruits that prevented bird feeding (Fig. 2). Most species that had large diaspores (*n* = 11) and were fed on by flying foxes were also dispersed by pigeons, while species with extra large diaspores (*n* = 6) were occasionally dispersed by rats or crabs (*n* = 4). Interestingly, crab-dispersed species were recorded only in this largest category (Fig. 2). Rats often consumed the same species as flying foxes, but for the majority of these, the rats destroyed the diaspores (Fig. 2). Overall, pigeons dispersed most of the species dispersed by flying foxes, but they could not handle the largest seeds, for which terrestrial animals were the only alternative dispersers (Fig. 2).

### Quantitative contributions of frugivores

**Figure 3.**
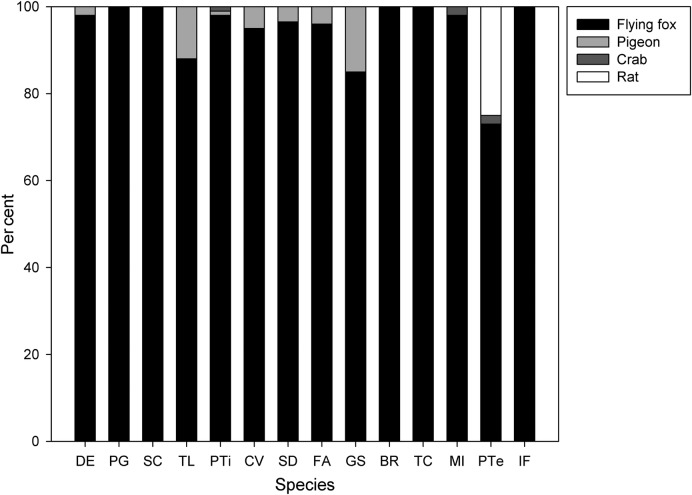
Percentage of seeds dispersed by different animal species for 14 plant species commonly consumed by flying foxes (see Table 1 for species identity). Plant species are arranged according to increasing diaspore size.

Although most plant species had multiple potential dispersers, flying foxes were the predominant dispersers for 14 species they commonly consumed, being responsible for >90 % of the dispersed diaspores for 12 species and >70 % for the remaining 2 (*n* = 83 individual plants in total) (Fig. 3). We recorded pigeon dispersal for six of these species, but rates approached 10 % for only two. Crabs and rats were each recorded two times, and rats dispersed a relatively high proportion of *Pandanus tectorius* diaspores.

### Distance distribution of dispersed seeds

**Figure 4.**
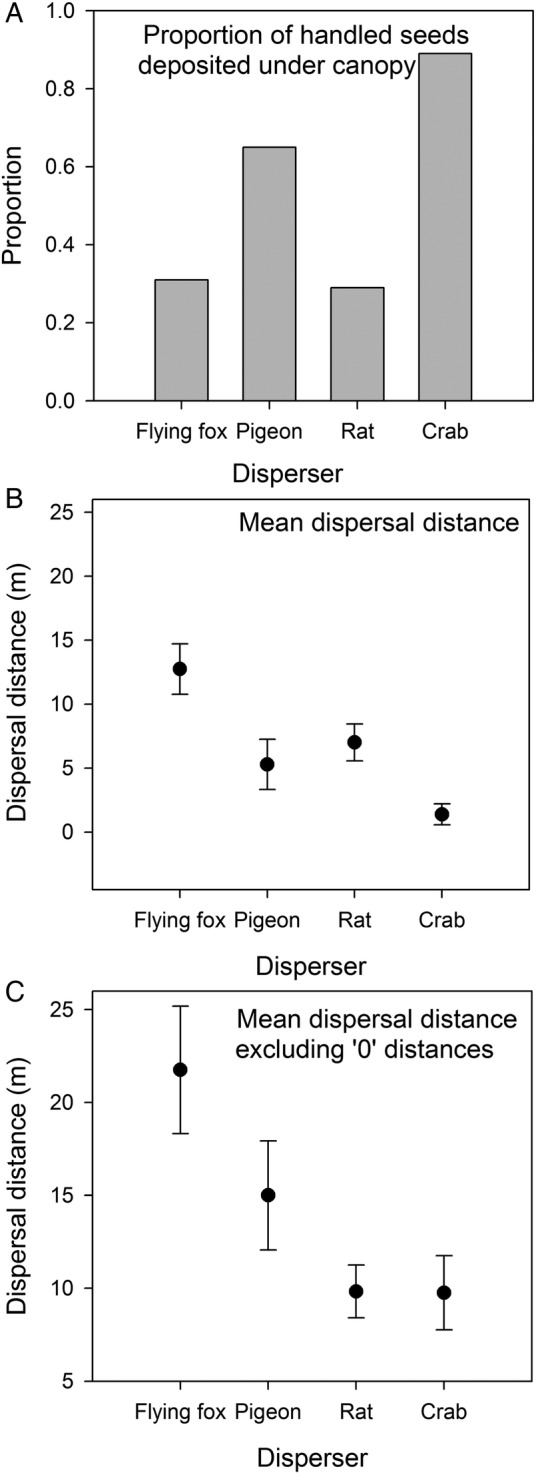
Summary of seed dispersal distances achieved by different dispersers. (A) Proportion of handled seeds deposited under the canopy of parent plants by each disperser. (B) Mean seed dispersal distances with 95 % confidence intervals. All handled seeds are included in this calculation, including those dispersed under parent crowns. (C) Mean distances (with 95 % confidence intervals) across all handled seeds that were dispersed away from the parent crowns (i.e. seeds dispersed 0 m are excluded). For both (B) and (C), values with non-overlapping confidence intervals are significantly different.

Flying foxes and rats deposited the lowest proportion of handled diaspores under conspecific canopies (31 % (*n* = 45 plants) and 29 % (*n* = 37), respectively), and there was no statistical difference between them (*Z* = −1.05, *P* = 0.15) (Fig. 4A). Both pigeons (*n* = 8) and crabs (*n* = 10) were significantly more likely to deposit handled diaspores under conspecific canopies than flying foxes were (65 and 89 %, respectively) (pigeons: *Z* = −2.64, *P* = 0.004; crabs: *Z* = −2.81, *P* = 0.002). If all handled diaspores are included (with those deposited under conspecific canopies), flying foxes dispersed diaspores a mean distance of 12.7 m from the canopy edge, which is significantly further than all other dispersers (the 95 % confidence intervals do not overlap) (Fig. 4B). Crabs deposited diaspores very close to conspecific crowns on average, while mean dispersal distances for pigeons and rats were intermediate (Fig. 4B). If only seeds dispersed away from conspecific canopies are considered, flying foxes still dispersed seeds significantly further than all other dispersers, followed by pigeons (Fig. 4C).

### Seed dispersal effectiveness and functional extinction

**Figure 5.**
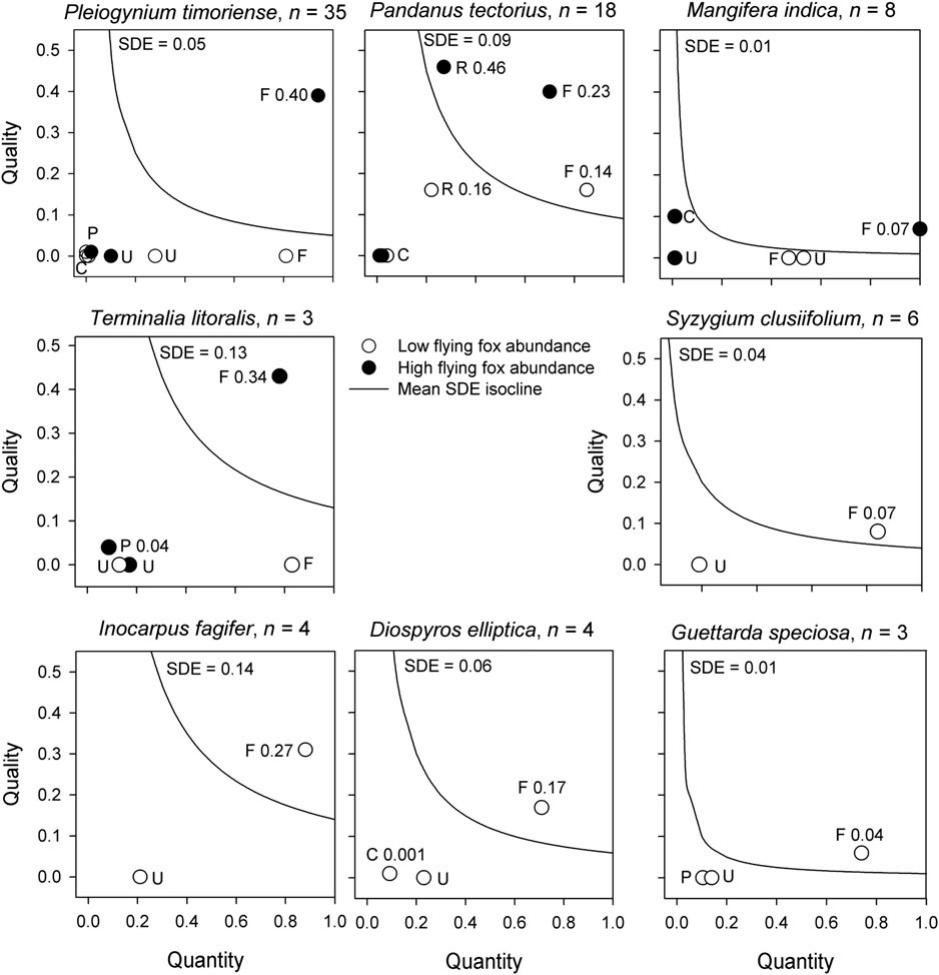
Seed dispersal effectiveness (SDE) of flying foxes and other seed dispersers for eight species commonly consumed by flying foxes. The figure shows the quantity (*x*-axis, proportion of seeds dispersed) and quality (*y*-axis; proportion of seeds moved at least 5 m from the canopy edge) components of SDE. The average SDE is indicated by the isocline (and value is noted). Animals above the isocline have greater-than-average effectiveness. Seed dispersal effectiveness values for independent animal species (F, flying fox; P, pigeon; C, crab; R, rat) and uneaten fruit (U) are written next to their label. Animals with SDE not shown had SDE = 0, since no seeds were dispersed away from the crown.

Flying foxes were the only effective seed disperser for seven of the eight species for which we had data for three or more individual plants (Fig. 5). For these plants, flying foxes displayed a greater-than-average effectiveness (their SDE value is above the isocline representing average SDE) and all other animals that handled fruit had SDE values at or near zero. The exception was *P. tectorius*, which was also dispersed by introduced rats.

The SDE of flying foxes was greater in sites where they were abundant than in sites where they were not (*t* = 3.13, df = 4, *P* = 0.0096; high abundance, mean ± 1 SD, 0.25 ± 0.13; low abundance, 0.05 ± 0.09). When flying foxes were abundant (relative to food plant availability), they generally produced high-quantity and high-quality components of seed dispersal (i.e. they dispersed many seeds and dispersed them away from the parent canopy). However, they were low-quality dispersers of the extra large, non-native *Mangifera indica* (Fig. 5). At low abundance, flying foxes still consumed relatively large amounts of fruit (high quantity) but dispersed little away from the crown (low quality). This pattern was confirmed statistically for one species (which had sufficient individuals to test); for *P.timoriense*, there was no difference in the quantity of seeds handled by flying foxes in low- and high-abundance sites (*t* = 0.06, df = 33, *P* = 0.47), but there was a difference in the quality of seed dispersal (i.e. proportion of seeds dispersed away from conspecific crowns; *t* = −2.38, df = 33, *P* = 0.011). Alternative frugivores did not compensate for this reduced dispersal, probably partly due to the fact that more fruit were not generally available for consumption in the absence of flying foxes.

**Figure 6.**
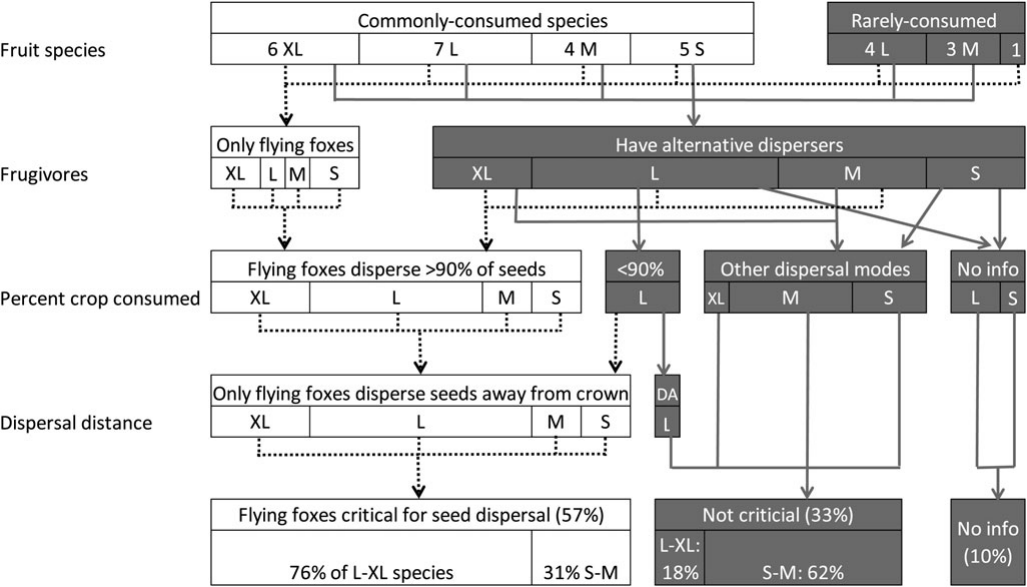
Percentage of fruit species consumed by flying foxes that are potentially dependent on them for seed dispersal. Species with small (S), medium (M), large (L) and extra large (XL) diaspores are indicated separately. Width of bars is proportional to the number of species represented within it. ‘Fruit species’ shows the number of species that are common or rare in the flying fox diet. ‘Frugivores’ indicates the proportion of species that have or lack an alternative disperser. ‘Percent crop consumed’ distinguishes those species for which alternative frugivores make almost no contribution (bats disperse >90 % of seeds), those species studied for which flying foxes dispersed <90 % of diaspores and those species that were not studied but which show features more consistent with bird or water dispersal. ‘Dispersal distance’ indicates whether flying foxes are the only disperser moving seeds away from the canopy or whether other species also contribute (DA, dispersal away).

Flying foxes are potentially critical for at least 57 % of their 30 food species (Fig. 6). We considered them not critical for 33 % of species because alternative dispersers existed that could handle at least 10 % of the available seeds, some of which would be dispersed beyond the parent crown. This category also includes species with fruit characteristics suitable for bird dispersal. We lacked sufficient information to evaluate three consumed species. Flying foxes were potentially critical for 76 % of the species with L or XL diaspores (>14 mm) and 31 % of species with M or S diaspores.

## Discussion

Flying foxes are essential components of the frugivore community in Tonga, and probably in many other island ecosystems, because they fulfil a non-redundant role in seed dispersal, especially for large-seeded plants. Although both flying foxes and Pacific pigeons (McConkey *et al*. 2004*a*) ate a diverse range of plant species, suggesting that they might be diet generalists, this was not reflected in a significant overlap in their roles as seed dispersers. Pacific pigeons are the only other major disperser of large seeds (Meehan *et al*. 2002; McConkey *et al*. 2004*a*), but they consume <40 % of the species dispersed by flying foxes and disperse very few seeds of these species. Elsewhere in the tropical Pacific, pigeon species are perhaps equally important as flying foxes where they overlap, but for a different subset of the available species, especially those with smaller seeds (Meehan *et al*. 2002, 2005; Fall *et al*. 2007). Crabs and rats are primarily considered seed predators (O'Dowd and Lake 1991; Green *et al*. 1997; McConkey *et al*. 2003; Lindquist and Carroll 2004; Pérez *et al*. 2008) but were capable of providing effective seed dispersal for some of the species dispersed by flying foxes. Flying foxes have been proposed as ‘keystone’ species in the tropical Pacific because of their potential importance as seed dispersers and pollinators (Cox *et al*. 1991; McConkey and Drake 2002; Scanlon *et al*. 2014). Our results confirm this status based solely on their seed dispersal function, with 57 % of all species consumed by flying foxes reliant on them for seed dispersal (76 % of larger-seeded species).

In island systems with low functional redundancy, seed predators may assume important roles as seed dispersers. In our study system, flying foxes damaged the seeds of two species for which we could identify no functional seed disperser, and granivorous rats and crabs were often the only alternative consumer of some species. Rats can disperse very small seeds internally (Williams *et al*. 2000; Shiels and Drake 2011), but frequently carry larger-seeded fruit to husking stations for processing, where some seeds may be abandoned and germinate (McConkey *et al*. 2003; Shiels and Drake 2011). Crabs were more likely than rats to disperse seeds (Lee 1985; Krishna and Somanathan 2014), but still destroyed seeds of three of the seven species we identified as crab-consumed. However, partial consumption of a seed's cotyledons does not always result in seed death. Provided the seed retains an intact embryonic axis, seed germination is possible and germination speed can be enhanced (Dalling *et al*. 1997; Takakura 2002; Pérez *et al*. 2008). Tolerance to cotyledon damage increases with seed size (Mack 1998). This was the only potential mode of seed dispersal for one common plant species (*Maniltoa grandiflora*) whose large seeds were consumed by both flying foxes and rats. Three plant species dispersed by flying foxes had multi-seeded diaspores that could be efficiently dispersed by seed predators. Rats removed the diaspores from the vicinity of the parent crown, but frequently destroyed only some seeds, leaving the remainder viable (D. R. Drake and K. R. McConkey unpubl. data).

An important difference in the seed dispersal capabilities of flying foxes compared with the more sedentary rats and crabs is their respective abilities to disperse seeds over long distances. The large-seeded species that might rely on any of these animals for dispersal are not carried passively in the gut of the animal, but rather actively in the mouth (or pincers). Flying foxes are highly mobile and may carry seeds as far as 10 km (Shapcott 1998), although shorter distances are more common. Flying foxes are the only means by which some of the large-seeded (particularly, XL) plant species may regularly reach another island; we recorded a bat-handled *Terminalia litoralis* seed that had no conspecific tree on the island and must have been carried at least 1.8 km. Without flying foxes, these long-distance dispersal events will not occur, except for coastal species that have buoyant seeds or can float by ‘rafting’ (Fall *et al*. 2007). Even local dispersal events are dominated by flying foxes in our study system. Crabs move fruits away from the source to avoid competition with other crabs (Lee 1985), and whereas distances are <10 m from parent crowns, they may leave seeds in burrows where the seed is protected from rodent predation (Lee 1985; Krishna and Somanathan 2014). Similarly, rats carry seeds to areas nearby where they can be sheltered from predators while feeding (McConkey *et al*. 2003), usually resulting in short dispersal distances (but sometimes up to 20 m). Given the low plant species diversity on these islands (Franklin *et al*. 2006) and the often close spacing of conspecifics, these distances may be adequate for escaping distance-dependent mortality (Chimera and Drake 2011; Comita *et al*. 2014) but may not be as effective in reaching gaps and enhancing gene flow as the more scattered dispersal patterns achieved by flying foxes are.

Contemporary ecosystems that persist on the islands in the tropical Pacific have a mélange of fruit-eating animals and fruits with varied origins and novel interactions (Steadman 1993; Shiels and Drake 2011; Spotswood *et al*. 2012). Losses in functional redundancy associated with disperser extinction or extirpation may have been partially supplemented by animal introductions (Schlaepher *et al*. 2011). Archaeological evidence indicates that the pre-human frugivore assemblage in Tonga was more diverse than today's, with two flying fox species and three large pigeon species (Steadman 1993, 2006; Meehan *et al*. 2002). Associated with the loss of some species has been the establishment of possibly one pigeon species (*D. pacifica*, Steadman 1993; Koopman and Steadman 1995) and three rat species. Although the rats may be functioning primarily as seed predators, they also provide a potentially important backup dispersal system for some species—particularly those with multi-seeded diaspores or with seeds that can germinate after partial damage (Pérez *et al*. 2008).

Island ecosystems that are dependent on flying foxes require not merely enough animals to maintain a viable population, but sufficient numbers for them to continue to disperse seeds and sustain the forests they ultimately depend on. Flying foxes become functionally or ecologically extinct as dispersers before their numbers are low enough to be considered ‘rare’ (McConkey and Drake 2006). At sites where they were functionally extinct, flying foxes continued to consume significant quantities of fruit, but dropped all—or almost all—seeds under the parent crown (McConkey and Drake 2006) where they may suffer higher mortality (Chimera and Drake 2011; Comita *et al*. 2014). Alternative frugivores did not compensate for this reduced seed dispersal role of flying foxes; while this may have been partly due to the fact that flying foxes continued to consume many fruit (making them unavailable to other consumers), the presence of fallen, unconsumed fruit under canopies suggest that these plant species are not heavily fed on by other frugivores regardless of flying fox density. This confirms the lack of redundancy in the seed dispersal network in our study system.

The Tongan flying fox that was the focus of our study is considered to be declining on some Pacific Islands (e.g. Cook Islands, Cousins and Compton 2005), and maintaining stable populations on others (e.g. Fiji, Scanlon *et al*. 2014), while its status remains unclear in most regions (Hamilton and Helgen 2008). A population decline of 80 % was caused by a cyclone that occurred after our study (McConkey *et al*. 2004*b*); it is not known to what extent the population has recovered, although it should be fairly robust to these periodic disturbances, provided hunting is not significant. Ongoing population monitoring is essential to ensure that this flying fox species, and others, can continue to perform their keystone roles in seed dispersal and possibly other ecological functions.

## Conclusions

In many simple island communities, bats are a dominant provider of ecosystem services. Two flying fox species disperse or pollinate nearly 80 % of canopy-trees in Samoa (Banack 1998), and bat species in Fiji serviced 42 % of plant species that were important to local communities (Scanlon *et al*. 2014). A single species of flying fox, *P. tonganus*, may disperse at least 50 % of the overstorey tree species in Vava‘u (Fall *et al*. 2007). The same study found that 77 % of the plants were adapted for bird dispersal, but with eight extant fruit-eating birds in this archipelago, this guild probably has more functional redundancy. Although overlap exists in the diets of flying foxes, birds and other dispersers, our study shows that this rarely translates into redundancy in the dispersal service provided by flying foxes, and more than half of the fruit species they consume can depend on them for dispersal. Given that the functional role of flying foxes in seed dispersal (and possibly pollination) can be severely affected by population decline (McConkey and Drake 2006), and that many island populations of *Pteropus* are already threatened, maintaining existing populations is very important. This is a difficult task given the often negative perceptions local agricultural communities have towards flying fox populations, owing to the losses of crops attributed to them (Scanlon *et al*. 2014). Promoting increased awareness of the important, and very vulnerable, roles of flying foxes in maintaining forests is potentially the most important step to ensure maintenance of the unique ecosystems in which they occur.

Island communities are inherently low in diversity, and the lack of redundancy we found in this simple island system may be typical of islands. The loss of a range of animal species, from reptiles (Hansen *et al*. 2008; Traveset *et al*. 2012) to birds (Caves *et al*. 2013), has been shown to have profound consequences for seed dispersal processes on islands, whereas the roles of similar species within mainland habitats have often gone unnoticed (Moura *et al*. 2015). Low ecological redundancy may characterize many island ecosystems, and this is likely to extend to other interactions as well, such as predation (Rogers *et al*. 2012), pollination and herbivory. In fact, pollination has been shown to be even more vulnerable in some island systems than seed dispersal (Kelly *et al*. 2010; Anderson *et al*. 2011). Consequently, identifying the critical species within island ecosystems across a range of ecological interactions is imperative for conservation.

## Sources of Funding

Our work was funded by the Wildlife Conservation Society (USA), Victoria University of Wellington (New Zealand), Percy Sladen Memorial Trust (UK) and Polynesian Airlines (Samoa).

## Contributions by the Authors

Both authors conducted fieldwork and were involved in the writing of the manuscript.

## Conflict of Interest Statement

None declared.
